# Subgroup J avian leukosis virus infection of chicken dendritic cells induces apoptosis via the aberrant expression of microRNAs

**DOI:** 10.1038/srep20188

**Published:** 2016-02-01

**Authors:** Di Liu, Manman Dai, Xu Zhang, Weisheng Cao, Ming Liao

**Affiliations:** 1 College of Veterinary Medicine, South China Agricultural University, Guangzhou, People’s Republic of China; 2 Key Laboratory of Veterinary Vaccine Innovation of the Ministry of Agriculture, Guangzhou, People’s Republic of China; 3 South China Collaborative Innovation Center for Prevention and Control of Poultry Infectious Diseases and Safety of Poultry Products, Guangzhou, People's Republic of China

## Abstract

Subgroup J avian leukosis virus (ALV-J) is an oncogenic retrovirus that causes immunosuppression and enhances susceptibility to secondary infection. The innate immune system is the first line of defense in preventing bacterial and viral infections, and dendritic cells (DCs) play important roles in innate immunity. Because bone marrow is an organ that is susceptible to ALV-J, the virus may influence the generation of bone marrow-derived DCs. In this study, DCs cultured *in vitro* were used to investigate the effects of ALV infection. The results revealed that ALV-J could infect these cells during the early stages of differentiation, and infection of DCs with ALV-J resulted in apoptosis. miRNA sequencing data of uninfected and infected DCs revealed 122 differentially expressed miRNAs, with 115 demonstrating upregulation after ALV-J infection and the other 7 showing significant downregulation. The miRNAs that exhibited the highest levels of upregulation may suppress nutrient processing and metabolic function. These results indicated that ALV-J infection of chicken DCs could induce apoptosis via aberrant microRNA expression. These results provide a solid foundation for the further study of epigenetic influences on ALV-J-induced immunosuppression.

Avian leukosis viruses (ALVs) are a group of avian retroviruses that induce tumors in chickens^1^. Chicken ALVs are classified into six subgroups (A-E and J) on the basis of the envelope glycoprotein responsible for viral interference patterns, virus neutralization, and host range^2^,^3^. In recent decades, ALV-J has become epidemic, emerging in commercial layers and local breed flocks throughout China^4^. ALV-J, which mainly induces myeloid leukosis, causes more serious damage than other virus subgroups^5^. Many recent studies have found that ALV-J and other subgroups are important coinfection factors in avian diseases^6^,^7^,^8^. ALV infection with a concomitant enhanced secondary infection is likely the outcome of immunosuppression; however, the mechanism of this immunosuppression has not been clarified^9^.

Dendritic cells (DCs), which were identified in mouse spleen tissue by Steinman and Cohn in 1973 and named for their typical morphology^10^,^11^, are professional antigen-presenting cells of the immune system that have the unique capacity to initiate primary immune responses^12^,^13^,^14^. DCs can regulate the immune response, and these cells express many different pathogen recognition receptors, such as Toll-like receptors, which are helpful for antigen presentation^15^. These functions are dependent on DC maturation, which is regulated largely by microRNAs (miRNAs)^16^,^17^,^18^,^19^.

As a retrovirus, the ALV provirus can randomly integrate into the host genome, which may result in the deregulation of gene expression, especially the expression of various regulatory factors such as miRNAs^20^. The mechanism of ALV provirus integration is unclear; thus, ALV integration is often deemed random. However, some references have indicated that the provirus could highly integrate in specific regions upstream of some genes such as oncogenes (c-myc, c-erb, EGFR, TERT, ZIC1, MET and others)^21^,^22^. Thus, ALV integration could also regulate the expression of specific miRNAs by currently unknown mechanisms. The processes of virus infection and replication also stimulate cells and influence miRNA expression; ALV-J infection might disrupt DC maturation via miRNA expression and further influence the immune response.

In this study, we found that ALV-J was able to infect chicken DCs. Infection altered normal DC functions, including their rate of maturation, while inducing apoptosis and causing aberrant miRNA expression. These changes in DC function may further result in immunosuppression.

## Results

### Chicken DC culture

**Figure 1:**
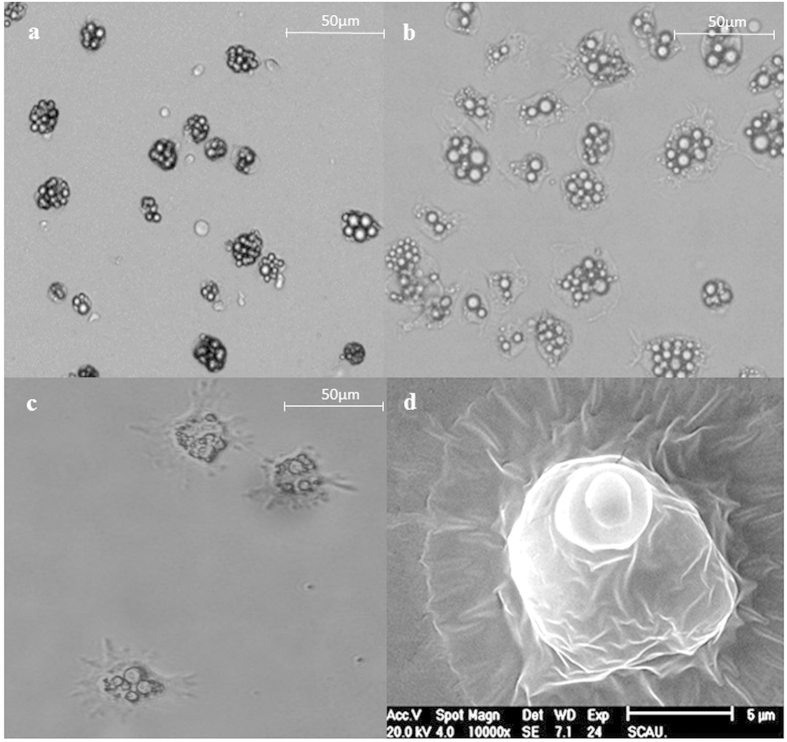
Morphology of DCs during differentiation. **(a)** Cell aggregates at days 2-3 (100× magnification). **(b)** The aggregates exhibited a dendritic appearance at days 6–8 (100× magnification). **(c)** The DCs exhibited typical morphology after LPS stimulation (200× magnification). **(d)** Mature DCs after LPS stimulation observed under scanning electron microscopy (10,000× magnification).

**Figure 2:**
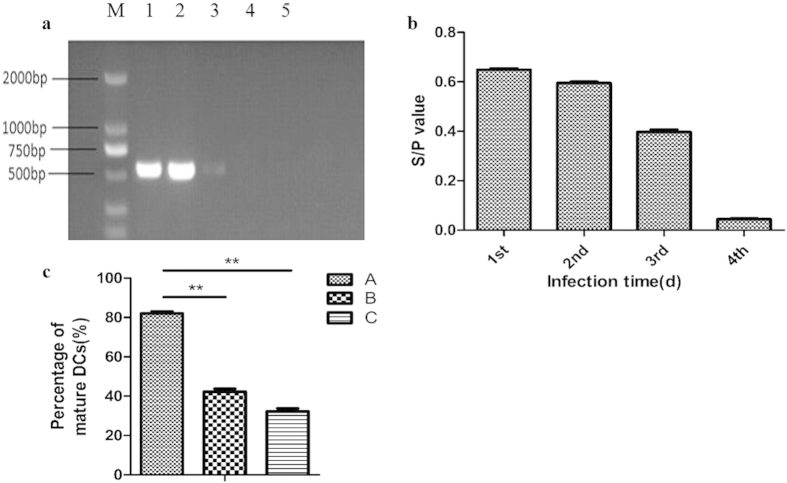
Effects of ALV-J infection on DCs. **(a)** An ALV-J-specific fragment (approximately 500 bp) was detected in infected DCs on days 1, 2 and 3. Lane M: DL2000 marker; lane 1: infected DCs on day 1; lane 2: infected DCs on day 2; lane 3: infected DCs on day 3; lane 4: infected DCs on day 4; lane 5: mock-infected DCs as a control. **(b)** ELISA for ALV-J antigens in DCs. S/P = (average OD of sample—average OD of negative control)/(average OD of positive control—average OD of negative control). An S/P value of >0.2 in DCs infected on days 1, 2 and 3 was considered to indicate positivity for ALV-J infection. An S/P value of <0.2 in DCs infected on day 4 was considered to indicate a lack of ALV-J infection. **(c)** Flow cytometric assay for DC maturation. A: The percentage of mature DCs among mock-infected DCs after stimulation with LPS was greater than 80%. B: The percentage of mature DCs among ALV-J-infected DCs after stimulation with LPS was approximately 40%. C: The percentage of mature DCs among mock-infected DCs without LPS stimulation was approximately 30%. **p < 0.01.

Mononuclear bone marrow cells are one type of stem cell that is capable of self-replication and differentiation into multiple cell types. Cell aggregate formation due to mononuclear cell proliferation and stimulation by cytokines increased from day 2 to day 4 (Fig. 1a). These aggregates sustained growth and differentiation, exhibiting a veiled or dendritic appearance in the subsequent 6–8 days (Fig. 1b). After the cells were stimulated with lipopolysaccharide (LPS) for 24 h on the tenth day of culture, most cells exhibited a dendritic structure, which indicates that the DCs are in the final stages of maturation (Fig. 1c). After the DCs were stimulated with LPS, their surface was rough, with many pleats and irregular structures protruding from the cell body (Fig. 1d). Flow cytometric analysis showed that more than 80% of the LPS-stimulated mature DCs were double positive for CD11c and CD86, whereas only 30% of the non-stimulated immature DCs had matured (Fig. 2c: A,C).

### DCs can be infected with ALV-J

The results of PCR and ELISA revealed that ALV-J could infect the cells on days 1, 2 and 3; this period is considered the early stage of cell differentiation in which bone marrow mononuclear cells form cell aggregates. The cell aggregates continued to develop new structures on their cell membrane and to divide after the third day.

The PCR results indicated that the proviral genome of ALV-J had integrated into the host genome of the DCs on the 1st, 2nd and 3rd days of infection (Fig. 2a). This integration is necessary for retrovirus replication. ELISA verified that ALV-J had completed its replication process in DCs that were infected on days 1, 2 and 3 (S/P > 0.2) (Fig. 2b).

Flow cytometric analysis showed that only 40% of infected DCs were double positive for CD11c and CD86 after LPS stimulation (infection time: day 2); this percentage was significantly less than that observed in the mock-infected DCs after LPS stimulation (*p* < 0.01) (Fig. 2c: B). These results revealed that the process of DC maturation was influenced by ALV-J infection.

### Analysis of DC apoptosis

**Figure 3:**
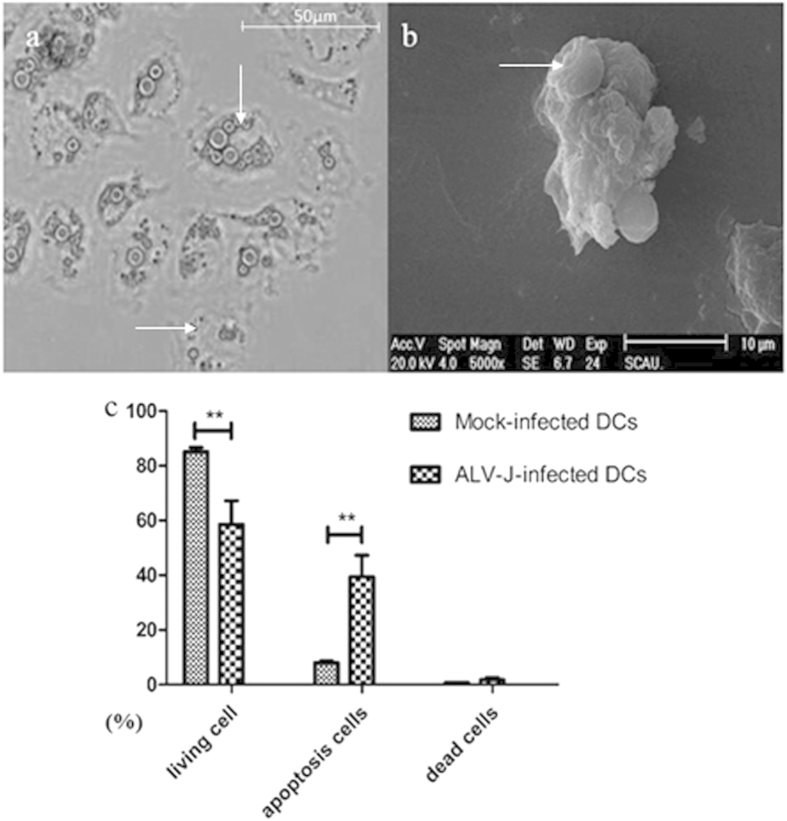
DC apoptosis assay. **(a)** Cell aggregates partially infected with ALV-J exhibited damage at days 6–8, with some cells missing from within the cell aggregates (arrow). **(b)** Crenation and apoptotic bodies were observed under scanning electron microscopy (arrow). **(c)** In mock- and ALV-J-infected DCs, the percentages of living cells were approximately 85% and 60%, respectively, whereas the percentages of apoptotic cells were approximately 8% and 40%, respectively. The percentage of apoptotic cells in ALV-J-infected DCs was significantly increased compared with that in mock-infected DCs. There was no significant difference in the percentage of dead cells. **p < 0.01.

Partial cell aggregates infected by ALV-J were damaged, with the aggregates demonstrating some cell loss on days 6-8 (Fig. 3a). The infected DCs appeared crenated under scanning electron microscopy, and obvious apoptotic bodies were present at the surface (Fig. 3b). The results of the annexin V-FITC assay revealed that the percentages of living and apoptotic cells (living cells undergoing apoptosis) were significantly different, although the percentage of dead cells was not significantly different (Fig. 3c). Approximately 40% of infected ALV-J DCs were undergoing apoptosis; this percentage was higher than the percentage of apoptotic cells in the mock-infected DC population.

### Alterations in miRNA expression in ALV-J-infected DCs

Analysis of the miRNA sequencing results revealed 122 miRNAs that were significantly differentially expressed between the control and infected DCs, indicating that many miRNAs in DCs were altered by ALV-J infection; among these miRNAs, 81 known and 41 novel miRNAs were identified. Of the 81 known miRNAs, 75 were significantly upregulated and 6 were significantly downregulated. Of the 41 novel miRNAs, 40 were significantly upregulated and 1 was significantly downregulated. Thus, most of the differentially expressed miRNAs were upregulated. The details of all significantly differentially expressed miRNAs are included in Supplementary Dataset 1.

### Verification of miRNA microarray results by qRT-PCR

**Figure 4:**
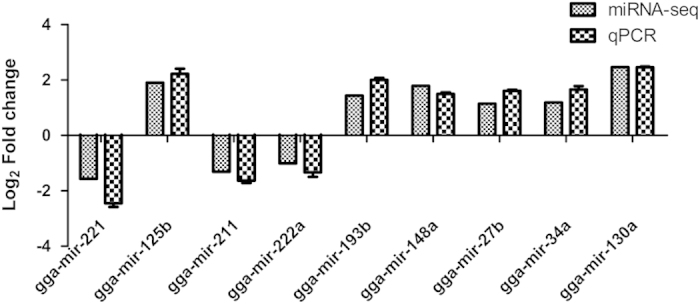
qRT-PCR confirmation of the miRNAs in ALV-J-infected DCs. Eight miRNAs were selected from the miRNA datasets and examined by qRT-PCR. All miRNA expression values were compared with the 5S endogenous control. The graph shows the consistency between the two methods in terms of upregulation or downregulation of expression.

The novel miRNAs for which no information could be found in any database were not analyzed in detail. As examining such a large number of miRNAs is difficult, eight miRNAs related to tumor formation and DC maturation were chosen for further verification. As shown in Fig. 4, gga-miR-221, gga-miR-125b, gga-miR-211, gga-miR-222a, gga-miR-193b, gga-miR-148a, gga-miR-27b, gga-miR-34a and gga-miR-130a were quantified by qRT-PCR, and these results were consistent with the sequencing analysis. Indeed, the qRT-PCR results demonstrated the same relative regulation of differentially miRNAs as that shown by the miRNA sequencing data.

### Gene Ontology (GO) analysis of the targets of the differentially expressed miRNAs

**Figure 5:**
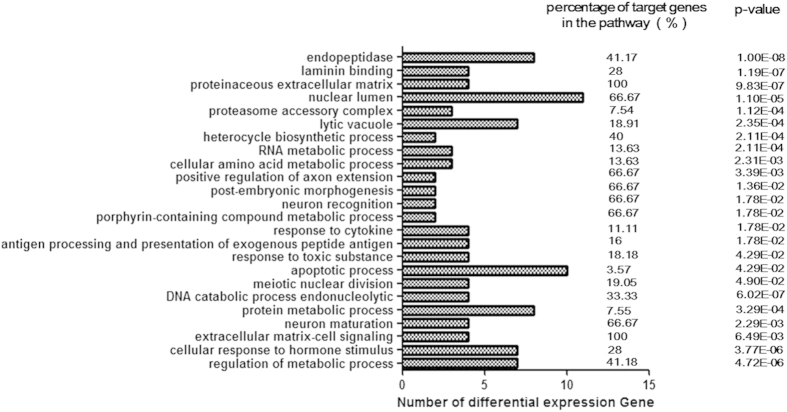
Enriched GO terms assay. Enriched GO terms for biological processes, molecular functions and cellular components of the target genes of differentially expressed miRNAs. The number and percentage of target genes are shown.

Target genes for the 81 known miRNAs were predicted by TargetScan software and used for GO analysis. The results of the functional analysis revealed significant enrichment of 24 GO terms (*p* < 0.05) (Fig. 5). Among these GO terms, “antigen processing and presentation of exogenous peptides” and “apoptosis” are related to the function of DCs and are consistent with the results of the apoptosis experiment. The target genes of gga-miR-204, gga-miR-211, gga-miR-221 and gga-miR-6651 are involved in the processes indicated by the two GO terms. The details of these GO terms are provided in Supplementary Dataset 2.

### KEGG pathway analysis of the targets of the differentially expressed miRNAs

To identify the biological pathways that might be associated with DC function, apoptosis and pathogenicity, known differentially expressed miRNAs were mapped onto signaling pathways for KEGG pathway analysis. The results revealed that 9 statistical categories were enriched significantly (*p* < 0.05) (Table 1). Three pathways of great interest were revealed: antigen presentation, apoptosis and chronic myeloid leukemia; the target genes of gga-miR-204, gga-miR-211 and gga-miR-6651 are involved in these pathways. The details of these pathways are provided in Supplementary Dataset 3.

## Discussion

Retroviruses can infect cells through special interactions between viral envelope proteins and cell surface receptors^23^. The protein chNHE1 was identified as a functional receptor for ALV-J; the expression of chNHE1 is likely ubiquitous in all chicken cells^24^. Furthermore, NHE1 is abundantly expressed in most tissues, leading to more effective targeting by ALV-J^25^. In this study, ALV-J was able to infect DCs on days 1–3 but not on day 4. During the first three days, bone marrow mononuclear cells formed aggregates without variation, and these cell aggregates further developed new membrane structures on day 4 likely as a result of cell differentiation. The protein structure of chNHE1 could be altered or damaged during the process of cell differentiation, which could explain the failure of ALV-J to infect the cells.

The effects of viruses on host cells vary and include, but are not limited to, cytocidal effects, stable infection and apoptosis. Most previous studies utilized chicken embryo fibroblasts (CEFs) and DF-1 cells, the two cell types used to produce ALV-J, whereas few studies have examined ALV-J infection in immune cells. Although no obvious cytopathic effects were reported in CEFs and DF-1 cells after ALV-J infection, we observed a different result for DCs, with ALV-J infection inducing DC apoptosis but not cytocidal effects. Thus, it is likely that ALV-J infection induced apoptosis in DCs. In addition, ALV-J disrupted DC differentiation *in vitro*.

The expression of a variety of genes is significantly altered by retroviral integration^26^, and viral long terminal repeats (LTRs) can activate host gene transcription^27^. Thus, viral integration near host genes may result in the deregulation of host gene expression^28^. As the tumorigenic mechanism of ALV has long been a primary focus, the cellular proto-oncogenes activated by ALV promoters through viral integration have been studied in depth^29^. However, because of this integration, any gene can potentially be enhanced or activated. Some miRNA expression could also be influenced by ALV-J integration. Cell differentiation occurs over a relatively long period, and ALV-J integration may be the major reason for the aberrant miRNA expression. Furthermore, ALV-J infection and replication in cells may influence miRNA expression. In this study, as many as 122 miRNAs were found to be significantly differentially expressed, and most of these miRNAs were upregulated. These results were different from previously reported results of miRNA expression in ALV-J-infected liver tissue and ALV-J-induced tumors^30^,^31^. In these two previous studies, the miRNAs gga-miR-221, gga-miR-211 and gga-miR-222a were found to be upregulated, whereas gga-miR-125b and gga-miR-193b were downregulated. These miRNAs play a key role in viral oncogenicity, as their overexpression accelerates tumor formation^32^,^33^. According to our data, gga-miR-221, gga-miR-211 and gga-miR-222a were downregulated and gga-miR-125b and gga-miR-193b were upregulated in infected DCs compared with mock-infected DCs. These opposite results reveal that different alterations in ALV-J-induced miRNA expression may occur in different cells and tissues. Thus, the outcome of ALV-J infection may directly depend on the cells or tissues studied. gga-miR-221, gga-miR-222, gga-miR148, gga-miR-27b, gga-miR-34a and gga-miR-130a, which are related to differentiation and maturation, could also further influence the function of mature DCs. Indeed, the processes of differentiation and maturation are disturbed by ALV-J infection, which may induce significant apoptosis in DCs^18^,^34^,^35^.

Systematic bioinformatic analysis of the differential expression of known miRNAs in ALV-J-infected DCs indicated that 24 GO terms were significantly enriched, including many related to cell biological processes. Based on these results, we found that antigen processing and presentation and apoptosis are significantly regulated by gga-miR-204, gga-miR-211, gga-miR-221 and gga-miR-6651. Subsequent KEGG analysis indicated that gga-miR-204, gga-miR-211 and gga-miR-6651 are involved in antigen processing and presentation, apoptosis and the chronic myeloid leukemia pathway. miRNAs negatively regulate gene expression through sequence-specific interactions with the 3’ untranslated regions of their target mRNAs, thereby causing translational repression or mRNA destabilization^36^. These four miRNAs were downregulated in infected DCs; thus, the expression of their targets was enhanced. The biological processes and pathways associated with antigen presentation, apoptosis and chronic myeloid leukemia, which are related to these miRNAs, may therefore be activated. Apoptosis was activated, as shown in the ALV-J infection experiment; however, none of the data indicated that the genes involved in apoptosis were directly activated by ALV-J integration. The reasons for apoptosis are various. Marrow, which contains many cell types, is susceptible to ALV. The chronic myeloid leukemia pathway was found to be activated in ALV-J-infected DCs; this activation might also occur in other cells in the marrow, and this pathway activation in different marrow cells might be related to the induction of myeloid tumors by ALV-J. As antigen processing and presentation target genes were not repressed in ALV-J-infected DCs, the live DCs infected with ALV-J might have retained certain antigen presentation functions.

The miRNAs with the greatest differential expression were upregulated after ALV-J infection, and these miRNAs repressed the transcription of their target genes, which are involved in various biological functions. The biological processes of nutrient conversion and energy metabolism were negatively affected by ALV-J infection. Because a balance between nutrient conversion and energy metabolism is required for the differentiation of DCs and other immune cells^37^, DC differentiation can be influenced by metabolic disorders induced by ALV-J infection. Cells that fail to differentiate into DCs gradually become apoptotic as the genes involved in apoptosis become activated by changes in abnormal cellular nutrient conversion and energy metabolism.

In many studies on ALV-J pathogenicity, immune tissue injury was deemed the major reason for immunosuppression^38^,^39^. In the present study, ALV-J was shown to influence the differentiation of DCs and to induce apoptosis though the aberrant expression of miRNAs *in vitro*. Because ALV-J is a retrovirus, hen-to-egg transmission of ALV may lead to the integration of viral genes into the host genome, potentially altering gene expression. ALV-J infection may also affect embryonic development and differentiation in a variety of cell types. The differentiation and maturation of DCs and other immune cells affect the immune response, and our results indicate that immunosuppression may occur earlier as a result of DC apoptosis before immune tissue injury. The chronic myeloid leukemia pathway was found to be activated, which might be related to myeloid tumors. These results require further research in vivo.

In conclusion, ALV-J infection was shown to interfere with DC differentiation and to induce aberrant miRNA expression and DC apoptosis, which resulted in abnormal antigen presentation and immune response suppression. This study suggests a possible mechanism by which ALV-J infection may cause immunosuppression.

## Materials and Methods

### Animals

Specific pathogen-free (SPF) chickens (less than 15 days old) were hatched from SPF eggs (Merial Vital Laboratory Animal Technology Company, Beijing, China). All chicken sampling procedures were approved by the Animal Care and Use Committee of South China Agricultural University (SCAU), and all animal research was conducted under the guidance of the SCAU Institutional Animal Care and Use Committee.

### Virus strains

The subgroup J avian leukosis virus strain NX0101 was kindly provided by Professor Cui of the College of Animal Science and Technology, Shandong Agriculture University, Taian, Shandong, China.

### Isolation and culture of chicken bone marrow-derived dendritic cells

BM-DCs were isolated and cultured according to a previously published protocol, with some modifications^40^. Briefly, mononuclear cells were isolated from the marrow of chicken femurs using chicken lymphocyte separation medium (Solarbio, Beijing, China). These cells were cultured in six-well plates containing RPMI-1640 complete medium (Gibco, CA, USA) with 10% heat-inactivated chicken serum (Gibco, CA, USA) at a final concentration of >1 × 10^6 ^cells/mL. And rh-GM-CSF and rh-IL-4 (Peprotech, NJ, USA) were also added at optimal concentrations (30~50 ng/mL). The medium was replaced with fresh medium every 2–3 days. The cells were cultured at 39 °C with 5% CO_2_ for 10 days, after which the cells were stimulated with 200 ng/mL LPS (Sigma, Santa Clara, CA, USA).

DCs were identified by morphological and flow cytometric analyses (FC500MCL/MPL, Beckman Coulter, USA). Flow cytometry was used to evaluate the cell surface expression of markers typically expressed on DCs. It is generally accepted that DCs exhibit high surface expression of CD11c and CD86^40^; thus, DC maturation was analyzed with a PE anti-mouse CD11c monoclonal antibody (mAb) and an FITC anti-mouse CD86 mAb (Affymetrix eBioscience, CA, USA).

### ALV-J infection of BM-DCs

We chose different time points during DC differentiation (days 1, 2, 3 and 4) for the virus infection experiments based on the durations of cell and virus cultures. DCs were infected with a dose of 10^4 ^TCID_50_/mL of the ALV-J NX0101 strain in serum-free medium, and the cells were incubated for 2 h at 39 °C with 5% CO_2_. DCs were also mock infected with serum-free 1640 medium alone under the same incubation conditions. After the medium (10% chicken serum, 50 ng/mL GM-CSF, 40 ng/mL IL4) was replaced, the infected cells were cultured at 39 °C with 5% CO_2_ for 7 days followed by stimulation with LPS for 24 h. The supernatants and cells were collected separately.

### ELISA and PCR

To detect ALV-J by ELISA, supernatants were collected from the cultures of infected DCs using an ALV antigen test kit (IDEXX, ME, USA). The IDEXX ALV antigen test kit is a commercial detection kit that is widely used in ALV antigen detection. Samples of ALV-J-infected and control DCs were tested, and S/P values were calculated according to the manufacturer’s instructions. S/P = (average OD of sample-average OD of negative control)/(average OD of positive control-average OD of negative control). An S/P value of >0.2 was considered positive for ALV-J infection, whereas an S/P value of  < 0.2 was considered negative for ALV-J infection.

cDNA was isolated from the DCs according to the manufacturer’s instructions (Omega Bio-Tek, Norcross, GA, USA), and PCR was performed using Taq DNA polymerase (New England Biolabs, Ipswich, USA) under the following conditions: 95 °C for 5 min, followed by 35 cycles at 95 °C for 30 s, 55 °C for 30 s and 72 °C for 1 min, with a final extension at 72 °C for 10 min. Primers (F: GGATGAGGTGACTAAGAAAG; R: CGAACCAAAGGTAAGAAAG) were designed based on the published target sequence of the ALV-J gp85 gene (GenBank: Z46390.1). The purified products were sequenced to verify target amplification.

### Maturation and apoptosis analysis of ALV-J-infected DCs

DCs were infected with a dose of 10^4^ TCID_50_/mL of the ALV-J NX0101 strain on day 2, and the infected cells were cultured for 7 days. The ALV-infected and mock-infected cells were collected after LPS stimulation for 24 h. The percentage of mature DCs in the infected cell population was also determined by flow cytometry with the PE anti-mouse CD11c and FITC anti-mouse CD86 mAbs. The percentage of apoptotic cells was determined using the Annexin V-FITC apoptosis detection kit (BestBio, Wuhan, China).

### High-throughput sequencing and analysis of miRNAs

DCs were infected with a dose of 10^4^ TCID50/mL of the ALV-J NX0101 strain on day 2, and the infected cells were cultured for 7 days. Two groups of samples (two biological replicates) of the ALV-J-infected and mock-infected DCs that had matured after LPS stimulation for 24 h were selected to further investigate the effects of ALV-J infection on these cells. These samples were processed for miRNA sequencing and analysis by the Beijing Genomics Institute (BGI), Shenzhen, China.

BGI used the Illumina HiSeq 2500/2000 platform for microRNA high-throughput sequencing, and tags were aligned to the miRBase database (http://www.mirbase.org/ftp.shtml) using BLAST to identify known miRNAs^41^. Novel miRNAs were predicted using miRDeep software^42^. After the miRNA sequencing results were obtained, BGI performed all of the bioinformatics analyses. The differential expression of miRNAs was analyzed by the DEGseq program, which is an R package for identifying differentially expressed genes from RNA-seq data (based on the R programming language software)^43^. The target genes of miRNAs were predicted using TargetScan and RNAhybrid software. We extracted the intersections or unions of target genes that were predicted by two software programs as the final prediction results.

### Quantitative real-time RT-PCR of miRNAs

Eight significantly differentially expressed miRNAs related to tumor formation and DC maturation were selected for confirmation. miRNA expression was analyzed by quantitative real-time PCR using an miRcute miRNA qPCR Detection Kit (Tiangen, Beijing, China) according to the manufacturer’s instructions. The cycling conditions for real-time PCR were as follows: 94 °C for 2 min, followed by 35 cycles of 94 °C for 2 s and 60 °C for 34 s. qPCR was carried out using a miRNA-specific forward primer (Table 2), and 5S RNA was used as an internal control. The reactions were performed using an ABI 7500 Real-Time PCR system (Applied Biosystems, Rotkreuz, Switzerland).

### GO and KEGG enrichment analysis

GO enrichment analysis provides all GO terms that are significantly enriched in a list of target genes of differentially expressed miRNAs. This method first maps all target genes to GO terms in the database (http://www.geneontology.org/), calculates the gene numbers for every term, and then uses hypergeometric tests to find significantly enriched GO terms in the input list of target genes based on ‘GO::TermFinder’ (http://smd.stanford.edu/help/GOTermFinder/GO_TermFinder_help.shtml/). We developed a strict algorithm to perform this analysis. The method used is described as equation “
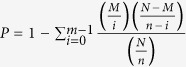
”, where N is the number of all genes with GO annotation, n is the number of target genes in N, M is the number of all genes that are annotated to certain GO terms, and m is the number of target genes in M. The calculated *p*-value then undergoes Bonferroni correction (*Encyclopedia of Measurement and Statistics*), taking a corrected *p*-value ≤0.05 as the threshold.

KEGG (http://www.genome.jp/kegg/) is used to perform pathway enrichment analysis of target genes. This analysis identifies significantly enriched metabolic pathways or signal transduction pathways in target genes compared with the whole genome background. The formula for this calculation is identical to that for GO analysis. The calculated *p*-value undergoes Bonferroni correction, taking a corrected *p*-value ≤0.05 as the threshold.

### Statistical analysis

Statistical comparisons were made using GraphPad Prism 5 software (GraphPad Software Inc., San Diego, CA), and statistical significance was represented by a *p*-value of <0.05 or 0.01.

## Additional Information

**How to cite this article**: Liu, D. *et al*. Subgroup J avian leukosis virus infection of chicken dendritic cells induces apoptosis via the aberrant expression of microRNAs. *Sci. Rep*. **6**, 20188; doi: 10.1038/srep20188 (2016).

## Supplementary Material

Supplementary Dataset 1

Supplementary Dataset 2

Supplementary Dataset 3

## Figures and Tables

**Table 1:** The predicted targets of the modulated miRNAs are involved in signaling pathways.

Pathway ID	KEGG pathway	Number of target genes	*p*-value
ko04612	Antigen processing and presentation	9	0.0005
ko04142	Lysosome	9	0.0068
ko04360	Axon guidance	12	0.0082
ko05146	Amebiasis	9	0.0103
ko04510	Focal adhesion	14	0.0178
ko04210	Apoptosis	6	0.0181
ko04974	Protein digestion and absorption	6	0.0465
ko05220	Chronic myeloid leukemia	6	0.0465
ko03050	Proteasome	3	0.0496

**Table 2:** Primers for miRNA RT-PCR.

miRNA	qRT-PCR forward primer
gga-miR-211	TTCCCTTTGTCATCCTATGCCT
gga-miR-125b	CTCCCTGAGACCCTAACTTGTGA
gga-miR-221	AACCTGGCATACAATGTAGATTTCTG
gga-miR-222b	GCTACATCTGGCTACTGGGTCTC
gga-miR-193b	CGTCAGCGGGGTTTTGG
gga-miR-148a	CGGAAAGTTCTGTGACACTCAGACT
gga-miR-27b	TCGGTTCACAGTGGCTAAGTTCT
gga-miR-34a	TGGCAGTGTCTTAGCTGGTTGTT
gga-miR-130a	TCGGATCGGCAGTGCAATAT
5S RNA	CTTAGCTTCCGAGATCAGACGAG
